# Distally Based Peroneus Brevis Flap for Primary and Salvage Coverage of Distal Leg and Ankle Defects in a Resource-Limited Setting: A Report of Three Cases

**DOI:** 10.7759/cureus.114645

**Published:** 2026-08-17

**Authors:** Khalid Abdel Aziz, Faris Abdon, Elbadawi Habib Allah, Mohamed ElAmin Salim

**Affiliations:** 1 Plastic and Reconstructive Surgery, Al Qassimi Hospital, Sharjah, ARE; 2 Plastic Surgery, Khartoum Teaching Hospital, Khartoum, SDN; 3 Medical Sciences Department/Medical Biochemistry Unit, Orotta College of Medicine and Health Sciences, Asmara, ERI; 4 Plastic Surgery, Armed Forces Hospital Southern Region, Khamis Mushait, SAU; 5 Plastic Surgery, Sharg Alneel Hospital, Khartoum, SDN

**Keywords:** ankle reconstruction, case series, distal leg trauma, limb salvage, local muscle flap, orthoplastic surgery, peroneus brevis flap, resource-limited setting

## Abstract

Distal leg and ankle defects with exposed bone are difficult to reconstruct, particularly after contaminated open injuries. Free tissue transfer is unavailable in many resource-limited hospitals, making reliable pedicled flaps essential. We report three adult men with Gustilo-Anderson type IIIB distal leg or ankle injuries treated with a distally based peroneus brevis muscle flap at a Sudanese teaching hospital. In the first patient, a 10×5 cm distal tibial defect after motorcycle trauma was covered immediately after debridement and external fixation, followed by skin grafting on postoperative day seven. In the second, a 7×5 cm lateral malleolar defect was covered on post-injury day four and grafted immediately. The third patient had a 13×6 cm donkey-bite/crush injury initially covered with a soleus flap; partial distal soleus flap loss re-exposed bone, and an adjunct peroneus brevis flap restored coverage on day six. All peroneus brevis flaps remained viable, all skin grafts took satisfactorily, and no clinically apparent deep infection or osteomyelitis was documented during six months of follow-up. All three limbs were salvaged. These cases show that the distally based peroneus brevis flap can provide both primary coverage and reactive salvage coverage for the selected distal leg and ankle defects when microsurgical reconstruction is unavailable.

## Introduction

Soft-tissue defects of the distal third of the leg and ankle are difficult to reconstruct because the local soft-tissue envelope is thin, exposed bone, tendon, or fixation hardware is common, and direct skin grafting over poorly vascularized structures is unreliable [1,2]. In open fractures, thorough debridement and timely vascularized soft-tissue coverage support fracture healing, reduce infection risk, and improve the prospect of limb salvage [2-4].

Free tissue transfer introduces healthy tissue from outside the zone of injury and remains important for major composite defects [2,5]. However, it depends on microsurgical expertise, operating microscopes, specialized instruments, suitable recipient vessels, and coordinated perioperative support. These resources are not routinely available in many resource-limited hospitals, where pedicled local flaps may be the only practical vascularized reconstructive option [5,6].

Soft-tissue defects of the distal third of the leg and ankle are difficult to reconstruct because the local soft-tissue envelope is thin, exposed bone, tendon, or fixation hardware is common, and direct skin grafting over poorly vascularized structures is unreliable [1,2]. In open fractures, thorough debridement and timely vascularized soft-tissue coverage support fracture healing, reduce the risk of infection, and improve the prospects for limb salvage [2-4].

Free tissue transfer introduces healthy tissue from outside the zone of injury and remains important for major composite defects [2,5]. However, it depends on microsurgical expertise, operating microscopes, specialized instruments, suitable recipient vessels, and coordinated perioperative support. These resources are not routinely available in many resource-limited hospitals, where pedicled local flaps may be the only practical vascularized reconstructive option [5,6].

Reconstructive options for distal leg and ankle defects include skin grafting when an adequately vascularized wound bed is present, local muscle flaps, reverse sural fasciocutaneous flaps, perforator-based propeller flaps, cross-leg flaps, and free tissue transfer [2,5]. The choice among these options depends on the defect size and location, exposed structures, local tissue viability, perforator availability, the zone of injury, surgeon expertise, and available reconstructive resources. Skin grafting is relatively simple but is unsuitable as definitive coverage for poorly vascularized exposed bone or fixation hardware [5]. Local muscle flaps provide vascularized tissue for smaller defects with exposed bone, tendon, or hardware. Still, their reach is limited, and their use depends on viable local muscle outside the zone of injury [2,5]. The reverse sural flap provides useful coverage of the distal tibia, heel, and malleolar regions without sacrificing a major lower-limb artery, although venous congestion is a recognized limitation [2,5]. Perforator-based propeller flaps can provide local fasciocutaneous tissue with low donor-site morbidity and good tissue matching while preserving functional muscle. Still, they require identification of a suitable perforator and careful operative planning, with a recognized learning curve [2,5]. Cross-leg flaps remain an option when suitable ipsilateral local or free-flap reconstruction is unavailable, but they require staged treatment and can make limb positioning and immobilization difficult [5]. Free flaps offer the greatest versatility for large or complex distal defects but require microsurgical expertise, appropriate recipient vessels, and specialized perioperative support [2,5].

The distally based peroneus brevis muscle flap is a recognized option for selected small-to-moderate defects of the distal leg, ankle, and hindfoot [1,6,7]. Its distal vascular basis depends on segmental perforators typically located 4-8 cm proximal to the lateral malleolus, permitting distal transposition without sacrificing a major leg artery [1,8,9]. Published series describe coverage of the lateral malleolus, distal tibia, Achilles region, and heel, with limited functional deficit when the peroneus longus is preserved [7-11].

Most available evidence is retrospective, and reports from clearly resource-limited settings remain uncommon [1,6,11]. The flap is also usually described as a stand-alone reconstructive option rather than as reactive salvage after another regional flap leaves inadequate distal coverage. Against this reconstructive spectrum, we aimed to describe the use of the distally based peroneus brevis flap in two clinically distinct roles: primary definitive coverage and reactive salvage after partial failure of another regional flap in a setting without routine microsurgical capability. The principal point of interest is therefore not the flap itself, which is well established, but its use as a reactive salvage adjunct after partial distal soleus-flap loss.

## Case presentation

Clinical setting and case overview

Three patients in this study were treated at Khartoum Teaching Hospital, Khartoum, Sudan, between January 1, 2021, and December 31, 2022. During this period, the public tertiary teaching hospital did not have routine access to an operating microscope, microsurgical instrumentation, or a dedicated microsurgical service. Clinical details were obtained from operative records, inpatient notes, radiographs, and outpatient follow-up documentation and were de-identified before manuscript preparation. The principal injury characteristics, management, and outcomes are summarized in Table 1.

**Table 1:** Clinical characteristics, management, and outcomes of the three patients.

Variable	Case one	Case two	Case three
Age and sex	25-year-old man	45-year-old man	60-year-old man
Mechanism	High-velocity motorcycle collision	Road-traffic friction injury	Donkey-bite/crush injury
Side	Right	Left	Left
Defect site	Distal third of leg	Lateral malleolus and ankle	Distal third of leg
Defect size	10×5 cm	7×5 cm	13×6 cm
Fracture	Comminuted distal tibia and fibula	Comminuted lateral malleolus	Comminuted distal tibia and fibula
Open-fracture grade	Gustilo-Anderson IIIB	Gustilo-Anderson IIIB	Gustilo-Anderson IIIB
Contamination and exposure	Dust and gravel; exposed tibial shaft	Road debris; exposed bone and periosteal denudation	Bite/crush contamination; exposed bone
Distal neurovascular status	Posterior tibial pulse palpable; sensation intact	Pulses palpable; sensation preserved	Distal perfusion preserved
First debridement	Same day	Same day	Same day
Initial antibiotics	Cefazolin and gentamicin	Cefazolin for 5 days	Cefazolin and gentamicin
Additional prophylaxis	Tetanus prophylaxis	Not applicable	Tetanus and rabies post-exposure prophylaxis
Fracture stabilization	External fixator	Below-knee backslab; no internal fixation	External fixator
Peroneus brevis flap role	Primary definitive coverage	Primary definitive coverage	Reactive salvage after partial soleus flap loss
Peroneus brevis flap timing	Day of admission	Post-injury day four	Day six
Additional flap used	None	None	Soleus flap at initial operation
Skin graft timing	Postoperative day seven	Immediate	Day 12
Flap and graft outcome	Viable flap; satisfactory graft take	Complete flap viability; 100% graft take	Both flaps viable; satisfactory graft take
Complications	None documented	Minor marginal granulation resolved with dressings	Partial distal soleus flap loss; no further complication after salvage
Hospital stay	12 days	5 days	20 days
Rehabilitation	Partial weight-bearing at 8 weeks; full weight-bearing at approximately 18 weeks	Protective-boot ambulation at 8 weeks; progressive weight-bearing	Gradual mobilization according to fracture healing
Follow-up	6 months	6 months	6 months
Final documented status	Limb salvaged; return to usual daily activities; no clinically apparent deep infection or osteomyelitis	Limb salvaged; durable coverage; return to usual daily activities; no clinically apparent deep infection or osteomyelitis	Limb salvaged; durable coverage; return to usual daily activities; no clinically apparent deep infection or osteomyelitis

Case 1

A 25-year-old man with no known comorbidities presented approximately two hours after a high-velocity motorcycle collision with a contaminated open injury to the distal third of the right leg. He had severe localized pain, inability to bear weight, active bleeding, and a wound contaminated with dust and gravel. Examination showed an irregular 10×5 cm wound with substantial soft-tissue loss, exposed tibial bone, deformity, swelling, ecchymosis, crepitus, and abnormal mobility. The posterior tibial pulse was palpable, capillary refill was acceptable, and sensation was intact. Radiographs demonstrated comminuted displaced distal-third fractures of the tibia and fibula, consistent with a Gustilo-Anderson type IIIB injury.

**Figure 1:**
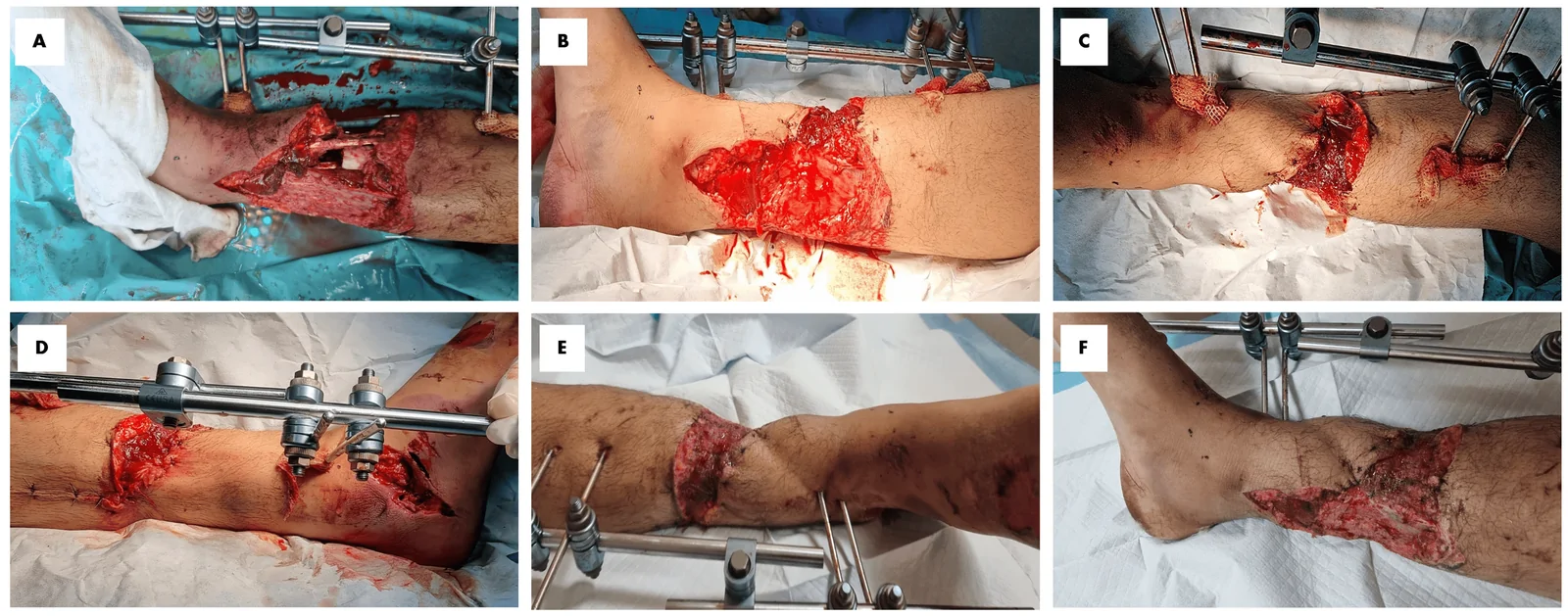
Immediate peroneus brevis flap coverage of a distal tibial defect. (A) Post-debridement wound with exposed tibial shaft and external fixation. (B-D) Transposition and inset of the distally based peroneus brevis muscle flap over the exposed tibia. (E,F) Viable, well-perfused flap before second-stage split-thickness skin grafting.

Intravenous cefazolin and gentamicin were started, and tetanus prophylaxis was administered. The same-day debridement and irrigation revealed extensive contamination, devitalized soft tissue, periosteal stripping, and exposure of the tibial shaft. The fracture was stabilized with an external fixator, and the exposed tibia was covered immediately using a distally based peroneus brevis muscle flap. The operative and early postoperative sequence is shown in Figure 1.

The limb was elevated, and flap viability was monitored clinically. After a healthy wound bed was confirmed, split-thickness skin grafting was performed on postoperative day seven. The flap remained viable, the graft took satisfactorily, and no further operation was required for soft-tissue coverage. The patient was discharged on day 12. Partial weight-bearing began at eight weeks, and full weight-bearing was allowed at approximately 18 weeks as the fracture healed. Serial clinical review, radiographs, and inflammatory markers when indicated showed no evidence of deep infection or osteomyelitis during six months of follow-up. At the six-month review, the limb was salvaged, and the outpatient record documented a return to usual daily activities.

Case 2

A 45-year-old man with no known comorbidities presented two hours after a high-energy road-traffic collision with a friction-type injury to the lateral aspect of the left ankle. Examination showed a 7×5 cm defect over the lateral malleolus with exposed comminuted bone, periosteal denudation, road-debris contamination, and surrounding nonviable friction-burned skin. Distal pulses were palpable, and foot sensation was preserved. The injury was classified as Gustilo-Anderson type IIIB.

**Figure 2:**
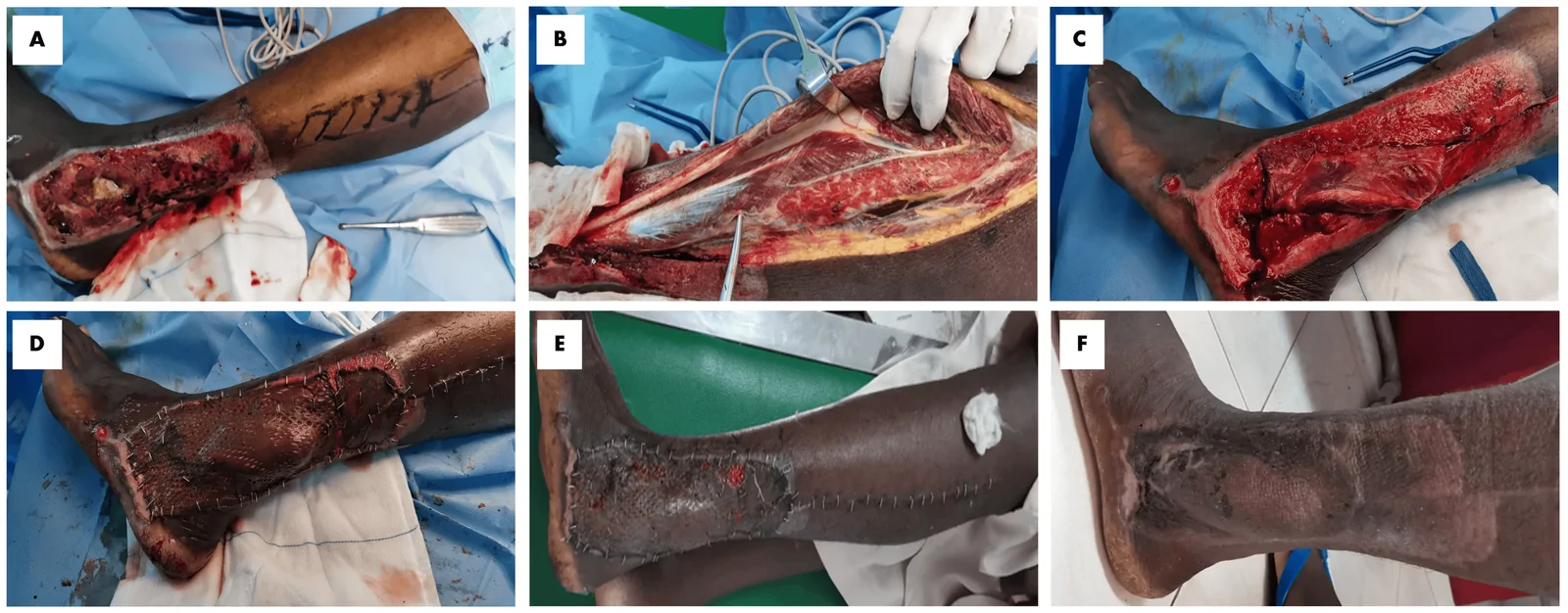
Peroneus brevis flap coverage of a lateral malleolar defect. (A) Debrided lateral malleolar defect with exposed bone. (B) Elevation of the distally based peroneus brevis muscle flap with preservation of the superficial peroneal nerve. (C) Distal transposition and inset before grafting. (D) Immediate appearance after split-thickness skin grafting. (E) Early healing with a viable flap and graft. (F) Durable late soft-tissue coverage.

A same-day irrigation and debridement were performed, and the limb was temporarily stabilized in a below-knee backslab. Intravenous cefazolin 2 g every eight hours was continued for five days; intraoperative wound cultures showed no growth. Clinical and radiographic assessment showed preserved tibiotalar congruity and a stable ankle mortise, so internal fixation was not undertaken. On post-injury day four, a distally based peroneus brevis flap was elevated with preservation of the superficial peroneal nerve and distal perforators identified approximately 6 cm proximal to the lateral malleolus. The muscle was inset over the defect and immediately covered with a 0.012-inch, 1:1.5 meshed split-thickness skin graft from the ipsilateral thigh. The donor site was closed primarily over a drain. The operative sequence and follow-up appearance are shown in Figure 2.

Postoperatively, the limb was elevated for 10 days, and low-molecular-weight heparin was administered for thromboprophylaxis. Assessment on postoperative day five showed complete flap viability and 100% graft take. Minor marginal granulation developed during the second postoperative week and resolved with dressings alone. The patient was discharged on postoperative day five, and protective-boot ambulation began at eight weeks, followed by progressive weight-bearing. At six months, the ankle remained clinically stable, coverage was durable, and the patient had returned to usual daily activities. No clinical evidence of deep infection or osteomyelitis was observed.

Case 3

A 60-year-old man presented several hours after a donkey-bite/crush injury to the distal third of the left leg sustained while working in a rural environment. The 13×6 cm wound had irregular margins, extensive devitalized soft tissue, contamination, exposed bone, deformity, and compromised surrounding skin. Distal perfusion was preserved. Radiographs demonstrated comminuted displaced distal-third fractures of the tibia and fibula, classified as a Gustilo-Anderson type IIIB injury.

**Figure 3:**
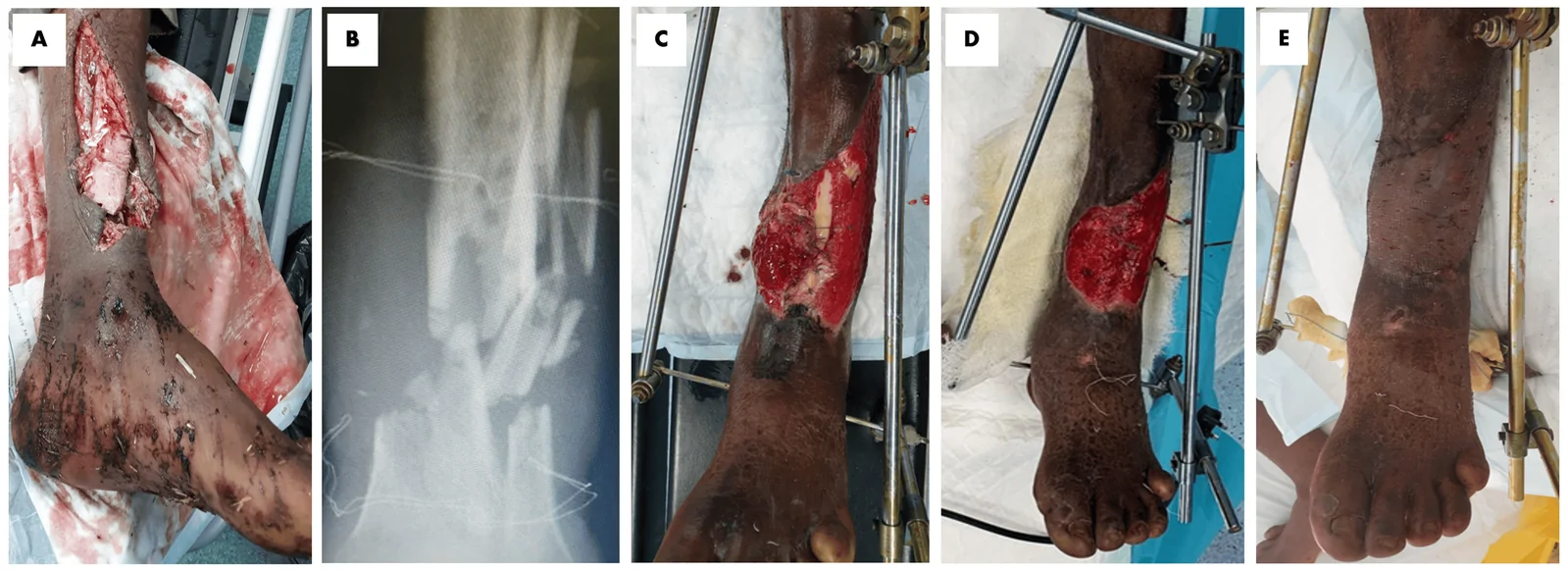
Salvage peroneus brevis flap after partial soleus flap loss. (A) Contaminated donkey-bite/crush wound at presentation. (B) Anteroposterior radiograph showing a highly comminuted displaced fracture of the distal tibia and fibula. (C) Residual distal defect with re-exposed bone after partial distal soleus flap loss. (D) Complete coverage after the inset of the adjunct distal-based peroneus brevis muscle flap alongside the surviving soleus muscle. (E) Stable coverage with a satisfactory split-thickness skin graft takes two weeks after discharge.

Intravenous cefazolin 2 g every eight hours and gentamicin 80 mg every eight hours, tetanus prophylaxis, analgesia, limb immobilization, and rabies post-exposure prophylaxis with vaccine and immunoglobulin were initiated. On the day of admission, aggressive debridement and irrigation were followed by external fixation and soleus muscle flap coverage. Partial distal loss of the soleus flap subsequently re-exposed bone. On day six, limited further debridement was performed, and a distally based peroneus brevis muscle flap was inset alongside the surviving soleus muscle, restoring complete distal coverage and providing a graftable wound bed. Split-thickness skin grafting was performed on day 12. The initial wound, radiograph, residual defect after soleus flap loss, adjunct flap coverage, and early graft result are shown in Figure 3.

Both muscle flaps remained viable, and the split-thickness skin graft remained viable and healed satisfactorily. The patient was discharged on day 20. At two weeks after discharge, the wound had stable coverage and satisfactory graft take. A six-month outpatient clinical and radiographic surveillance showed no evidence of deep infection or osteomyelitis. At six months, coverage remained durable, the limb was salvaged, and the outpatient record documented a return to usual daily activities. No additional bony procedure was documented during the six-month follow-up; fracture management continued under the orthopedic team with serial clinical and radiographic assessment.

## Discussion

These three cases illustrate two clinically distinct roles for the distally based peroneus brevis flap in a hospital without routine microsurgical reconstruction. It provided primary definitive coverage in two contaminated open injuries and reactive salvage coverage when partial distal loss of an initial soleus flap left bone exposed in the third. All peroneus brevis flaps remained viable, all split-thickness skin grafts remained viable and healed satisfactorily, and all three limbs were salvaged at six months. The contribution is not a new flap or proof of superiority but a practical demonstration that a familiar pedicled muscle flap can be used flexibly when free tissue transfer is not feasible.

Vascularized muscle coverage may benefit exposed bone by improving perfusion, filling dead space, and supporting the local biological environment of fracture healing [5,12]. The peroneus brevis flap also has useful anatomical characteristics: it is based on distal segmental perforators, can usually be elevated without sacrificing a major leg vessel, and reaches the distal third of the leg and ankle while preserving the peroneus longus [1,8,9]. In the second patient, distal perforators were identified approximately 6 cm proximal to the lateral malleolus, within the published anatomical range.

Perforator-based propeller flaps are an important alternative for selected small-to-moderate defects of the distal leg and ankle. They can provide like-with-like fasciocutaneous coverage while preserving functional muscle and avoiding sacrifice of a major axial vessel [2,5]. Their use, however, depends on identification of a suitable perforator outside the zone of injury, careful flap design and rotation, and appropriate surgical experience; a learning curve with these flaps is recognized [2,5]. Accordingly, reconstructive choice should be individualized according to defect size and location, exposed structures, the zone of injury, perforator availability, local tissue viability, surgeon expertise, and available resources, rather than considering either a propeller flap or a peroneus brevis flap as a universal first-line option. In the present patients, vascularized muscle coverage was selected for wounds with exposed bone in a setting where routine microsurgical reconstruction was unavailable.

The primary-coverage results in Cases 1 and 2 are consistent with published experience. A 2024 review of 222 patients reported an overall complication rate of 21% and a revision surgery rate of 7%, with skin graft loss among the common complications [1]. A series of 21 patients reported complete flap survival and healing by nine weeks, with one partial graft loss [7]. A series from a resource-challenged setting similarly supported the flap as a local alternative for patients who might otherwise require transfer for microsurgical coverage [6]. These reports support its use in selected small-to-moderate defects but do not justify treating it as a universal substitute for free tissue transfer.

The third patient provides the most distinctive lesson. Donkey bites can combine penetrating trauma, crush injury, and substantial bacterial contamination, with additional tetanus and rabies considerations in endemic settings [13,14]. After partial distal soleus flap loss, the peroneus brevis flap remained available and restored distal coverage without sacrificing the surviving proximal flap. The existing peroneus brevis literature more commonly describes stand-alone reconstruction [1,6,7]. This case therefore supports the flap as a reactive salvage option when wound evolution or partial failure of an initial regional flap leaves a residual distal defect. It does not establish that the flap should be prospectively reserved in every reconstructive plan.

The resource-limited setting is central to interpretation. Free tissue transfer remains appropriate for large composite defects, major vascular injuries, and wounds beyond the reach of local flaps [2,5]. At the study hospital, however, microsurgical equipment and a dedicated service were unavailable. In that context, a pedicled flap that preserves major vessels and can be raised with standard instruments may offer a realistic limb-salvage route [5,6]. This context-specific value should not be confused with comparative effectiveness.

Complications of the peroneus brevis flap are not negligible. Published problems more commonly include distal or marginal flap necrosis and skin-graft loss than complete flap failure [1,9,11]. A historical review reported an overall complication rate of 41.6%, whereas more contemporary data suggest lower rates, possibly reflecting improved patient selection and technique [1,11]. Careful assessment of defect size, perforator availability, the zone of injury, and the need for free tissue transfer remains essential.

This report has important limitations. It includes only three patients with heterogeneous injury mechanisms and no comparison group. Follow-up was limited to six months, which cannot rule out all late fracture-related infections or osteomyelitis. Validated functional scores, range-of-motion measurements, pain scores, and formal return-to-work data were not collected; return to usual daily activities was derived from routine outpatient documentation. Wound cultures were obtained only in the second patient, and late clinical photographs were unavailable for two patients. In addition, the Mangled Extremity Severity Score (MESS) was not recorded prospectively and could not be reliably reconstructed from the available retrospective documentation. The observations are therefore descriptive and hypothesis-generating.

## Conclusions

The distally based peroneus brevis muscle flap can be a practical local option for selected distal leg and ankle defects when free tissue transfer is unavailable. In this report, it provided primary coverage in two patients and reactive salvage coverage after partial soleus flap loss in a third. Its use should remain selective and should not be interpreted as a substitute for free tissue transfer when the latter is indicated and available. Larger prospective studies with longer follow-up and standardized functional outcomes are needed.

## References

[1] Mégevand V, Scampa M, Suva D, Kalbermatten DF, Oranges CM (2024). Versatility of the peroneus brevis muscle flap for distal leg, ankle, and foot defects: a comprehensive review. JPRAS Open.

[2] Zeiderman MR, Pu LL (2021). Contemporary approach to soft-tissue reconstruction of the lower extremity after trauma. Burns Trauma.

[3] Cao Z, Li C, He J, Qing L, Yu F, Wu P, Tang J (2022). Early reconstruction delivered better outcomes for severe open fracture of lower extremities: a 15-year retrospective study. J Clin Med.

[4] Dheenadhayalan J, Nagashree V, Devendra A, Velmurugesan PS, Rajasekaran S (2023). Management of open fractures: a narrative review. J Clin Orthop Trauma.

[5] Khundkar R (2019). Lower extremity flap coverage following trauma. J Clin Orthop Trauma.

[6] Antúnez M, Huyen C, Neiman R (2024). Pedicled peroneus brevis muscle flaps as an alternative to fasciocutaneous rotational flaps for lower-extremity soft tissue defects. J Orthop Trauma.

[7] Malahias M, Khalil H, Abdalbary SA, Abdelkader R (2020). Distally-based peroneus brevis turnover muscle flap in the reconstruction of soft tissue defects. Plast Reconstr Surg Glob Open.

[8] Vaienti L, Cottone G, Zaccaria G, Rampino Cordaro E, Amendola F (2022). One-step approach for infections after achilles tendon open repair: the distally based peroneus brevis muscle flap. Int J Low Extrem Wounds.

[9] Jose J, Parameswaran SC, Rajappan A, Prameela LN (2022). Surgical outcome of distally based peroneus brevis flap: a retrospective study. Cureus.

[10] Nava CM, Martineau J, Suva D, Kalbermatten DF, Oranges CM (2024). Distally based peroneus brevis flap: reconstruction of complex soft-tissue defects with bony infection of the lateral malleolus. J Plast Reconstr Aesthet Surg.

[11] Ensat F, Hladik M, Larcher L, Mattiassich G, Wechselberger G (2014). The distally based peroneus brevis muscle flap-clinical series and review of the literature. Microsurgery.

[12] Chan JK, Harry L, Williams G, Nanchahal J (2012). Soft-tissue reconstruction of open fractures of the lower limb: muscle versus fasciocutaneous flaps. Plast Reconstr Surg.

[13] Abrahamian FM, Goldstein EJ (2011). Microbiology of animal bite wound infections. Clin Microbiol Rev.

[14] Tiemdjo HG, Coulibaly T, Touré AA (2009). Paediatric open tibiofibular fractures following a donkey bite. A report of two cases. Orthop Traumatol Surg Res.

